# A comparative approach for the investigation of biological information processing: An examination of the structure and function of computer hard drives and DNA

**DOI:** 10.1186/1742-4682-7-3

**Published:** 2010-01-21

**Authors:** David J D'Onofrio, Gary An

**Affiliations:** 1 College of Arts and Science, Math Department, University of Phoenix, 5480 Corporate Drive, Suite 240, Troy, Michigan, 48098, USA; 2 Control Systems Modeling and Simulation, General Dynamics, 38500 Mound Rd, Sterling Heights, MI,48310, USA; 3 Department of Surgery, Northwestern University Feinberg School of Medicine, 676 North St Clair, Suite 650, Chicago, IL 60611, USA

## Abstract

**Background:**

The robust storage, updating and utilization of information are necessary for the maintenance and perpetuation of dynamic systems. These systems can exist as constructs of metal-oxide semiconductors and silicon, as in a digital computer, or in the "wetware" of organic compounds, proteins and nucleic acids that make up biological organisms. We propose that there are essential functional properties of centralized information-processing systems; for digital computers these properties reside in the computer's hard drive, and for eukaryotic cells they are manifest in the DNA and associated structures.

**Methods:**

Presented herein is a descriptive framework that compares DNA and its associated proteins and sub-nuclear structure with the structure and function of the computer hard drive. We identify four essential properties of information for a centralized storage and processing system: (1) orthogonal uniqueness, (2) low level formatting, (3) high level formatting and (4) translation of stored to usable form. The corresponding aspects of the DNA complex and a computer hard drive are categorized using this classification. This is intended to demonstrate a functional equivalence between the components of the two systems, and thus the systems themselves.

**Results:**

Both the DNA complex and the computer hard drive contain components that fulfill the essential properties of a centralized information storage and processing system. The functional equivalence of these components provides insight into both the design process of engineered systems and the evolved solutions addressing similar system requirements. However, there are points where the comparison breaks down, particularly when there are externally imposed information-organizing structures on the computer hard drive. A specific example of this is the imposition of the File Allocation Table (FAT) during high level formatting of the computer hard drive and the subsequent loading of an operating system (OS). Biological systems do not have an external source for a map of their stored information or for an operational instruction set; rather, they must contain an organizational template conserved within their intra-nuclear architecture that "manipulates" the laws of chemistry and physics into a highly robust instruction set. We propose that the epigenetic structure of the intra-nuclear environment and the non-coding RNA may play the roles of a Biological File Allocation Table (BFAT) and biological operating system (Bio-OS) in eukaryotic cells.

**Conclusions:**

The comparison of functional and structural characteristics of the DNA complex and the computer hard drive leads to a new descriptive paradigm that identifies the DNA as a dynamic storage system of biological information. This system is embodied in an autonomous operating system that inductively follows organizational structures, data hierarchy and executable operations that are well understood in the computer science industry. Characterizing the "DNA hard drive" in this fashion can lead to insights arising from discrepancies in the descriptive framework, particularly with respect to positing the role of epigenetic processes in an information-processing context. Further expansions arising from this comparison include the view of cells as parallel computing machines and a new approach towards characterizing cellular control systems.

## Background: A Case for Comparison

A biological cell can be viewed as a dynamic information-processing system that responds to and interacts with a varied and changing environment. Cellular actions rely on a set of operations between the genetic information encoded in the cell's DNA and its intracellular information-processing infrastructure (RNA and proteins). The structure and function of this information-processing complex are of great interest in the study of both normal cellular functions (such as differentiation and metabolism) and pathological conditions (such as oncogenesis and dysregulation). In order to better examine these complex behaviors it may be beneficial to identify the essential aspects of centralized information processing, and then seek analogous systems through which comparative analysis can be performed. Focusing on the interactions between cellular data and data processing can lead to a description of a cell as a biomolecular computer [1]. Alternatively, digital computers are highly-engineered information processing systems, and lessons drawn from computer science may provide a framework for comparison between an abstract description of the informational and computational elements of a cell and the architecture of a computer system [1,2]. Since the cell represents a level of complexity that is orders of magnitude greater than the most sophisticated computer system, caution must be exercised when making such analogies. However, the establishment of a mapping between the properties and functions of a biological cell and a digital computer may allow lessons learned from the design and engineering of computer systems to be transferred into the biomedical arena. This in turn can potentially lead to greater understanding of the dynamic processes and control mechanisms involved in gene regulation and cellular metabolism. Furthermore, the process of comparative analysis can be extended in an iterative process, such that mappings between cells and computers at one level may lead to insights for further mappings in terms of organization and structure.

A central common feature of both cellular and silicon systems is the existence of a dedicated and distinct centralized information storage and processing complex. In a digital computer, this complex is divided into hardware and software. We define the hardware as the physical components of the computer, along with the non-mutable design specifications/controllers of those physical components. Therefore, the hardware of a computer consists of the computer chip (also known as the central processing unit, or CPU) consisting of gates, registers and logic circuits, the actual disk of the hard-drive including the servo-mechanisms attached to the hard drive, RAM (Random Access Memory), ROM(Read Only Memory), controllers and I/O peripherals. The function of the CPU is intimately tied to its instruction set architecture (ISA), which defines how it will actually execute a program. We define software as the instruction set that tells the hardware how to implement computation and process information. Information in the form of software abstraction also includes the organization of that information, as opposed to a physical object. The software aspect of the centralized information-processing complex in a computer consists of the organization of its data, the rules for accessing, storing and processing its data (known as its Format), its operating system and its programs. It should be noted that these aspects of the computer's information processing complex are not intimately tied to the hardware, and can be altered and transferred from one computer to another.

Using these definitions, we consider the hardware of the cellular information-processing complex to be represented by its physical genetic material, gene expression machinery and the physical components of the cell (proteins, enzymes, etc.). The general architecture/spatial organization of the cell, and the effect of these spatial configurations on the manifestation of biochemical laws, can be viewed as similar to a computer's ISA [3]. The software aspect of the cell is represented in the informational content of its genome sequence (i.e. the specific pattern of nucleic acids). Those aspects of the DNA sequence that code for the structure and function of the molecular machinery of DNA replication, RNA transcription and protein assembly through translation, can be considered analogous to a computer's software instructions in relation to its basic input/output system (BIOS) and operating system. The field of molecular semiotics suggests that a cellular language exists for the instruction set for these cellular processes, and that this language in manifest in the sequence of the DNA [1]. The information within DNA consists of a quadruple genetic code consisting of Quad bits (Qbits) of the nucleotides of adenine (A), cytosine (C), thymine (T) and guanine (G) representing a base 4 system.

We propose that a cell's centralized information-processing complex, composed of its DNA and associated molecular machinery, can be considered analogous to a digital computer's hard drive (CHD) and operating system. This descriptive framework is established via comparative analysis between the architecture and function of the CHD and the structure and function of eukaryotic DNA, which we now define as the DNA hard drive (DHD). The computer ATA (Advanced Technology Attachment) hard drive will serve as a reference for the development of the comparative framework. The comparison will utilize the descriptions of functional equivalence between aspects of the CHD and the DHD, which is defined as follows:

When two systems (A and B) are to be compared to each other, they are said to be functionally equivalent if there is some minimal function that is intrinsic to system A, which can also then be identified in system B. If this functionality can be shown to exist in both systems then the systems are functionally equivalent even if they are physically different.

While many functions and operations characterize the CHD, its actions are described for the purposes of this comparison in terms of four major functional properties that are critical to centralized information processing. These four functional properties are:

(1) Orthogonal Uniqueness of Information. This refers to the property of information storage and representation that allows for unambiguous interpretation of the information when it is processed. Specifically, the property of orthogonality states that for any information system to represent its information in an unambiguous fashion there must be a one for one functional correspondence between the information and its physical manifestation.

(2) Low level formatting of information. This refers to the structure and organization of how information can be physically stored and subsequently accessed in a particular medium. It defines a relationship between the physical properties of the storage medium device and the configurations of those physical properties as the medium is imprinted with the information being stored.

(3) High level formatting of information. This refers to logical structures representing the organization of informational content/data of the system that is imprinted via the low-level formatting of the storage medium device. The goal of this level of organization is to optimize the efficiency and accuracy with which the stored information can be located, accessed and processed.

(4) Translation of stored information to usable information. This property refers to the mechanisms by which the information on the storage medium device is actually retrieved and passed to the rest of the information processing machinery, i.e. for the subsequent use of the information. It represents the necessary step for the utilization of stored information by the overall system. In a computer, this function is performed by the hard drive controller; in a cell, this process is highly complex, and involves the interplay of transcriptional and RNA interference complexes, splicosomes, microRNA's and post-transcriptional protein modifications.

This manuscript will proceed in four sections: 1) initial description of these four properties as manifest in the CHD, 2) identification of correlations and expansions to these properties by structural and informational aspects of the of DHD, 3) examination of the current discrepancies between the CHD and the DHD, and how these discrepancies may enhance our understanding of cellular information processing, and 4) concluding remarks with respect to the potential utility of this comparative approach.

## A computer hard drive (CHD) review: structure and function

**Figure 1:**
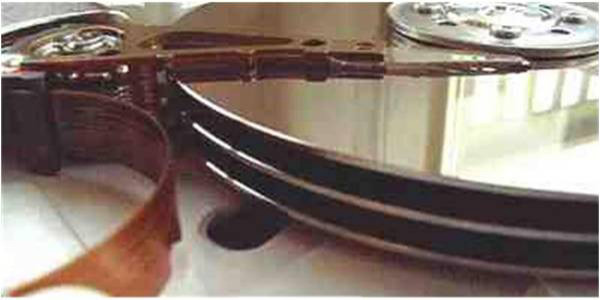
**Computer Hard Drive**. Computer Hard drive showing multiple disks and read/write head. Picture from "How things Work" by Marshall Brain.

The CHD is the central storage unit for information pertaining to the data, programs and operating systems that govern digital computers. Modern hard drives can store over 1000 gigabytes (Gbyte) of coded information and this number is increasing as the technology further develops. Hard drives store information in the form of magnetized dipole regions of its disk containing magnetic lines of flux. The magnetic flux is both written to, and read from, a component known as the servo head. The servo head consists of both write and read devices co-located within the servo control mechanism. It moves radially across the hard disk until it reaches a preset position where it will either read or write information to the disk. There maybe one or more disks stacked on top of each other forming cylinders of information (see figure 1). For the purpose of this discussion, the cylinder will be ignored and the description simplified to a single disk. Binary information in the form of file systems or data is encoded onto the hard drive disk through the use of magnetic elements. A logical "1" is represented as flux lines traveling from north pole to south pole followed by a flux reversal. A logical "0" is represented as flux lines traveling from south pole to north pole followed by a flux reversal. Therefore the polarity of the flux lines determines whether you have a logical one or zero (the language of digital computers). The read portion of the servo head detects transitions in the magnetic flux between adjacent magnetized regions [4,5]. The flux information will be converted to electrical signals, which are interpreted by encoding/decoding algorithms as a logical one or zero, creating the binary information. The information regions on the disk are based solely on the transition of the boundary conditions characterized by a change in magnetic flux between adjacent regions. Robustness of the boundary condition (when properly arranged) is what makes the creation of binary information unique. The sensitivity of the read servo head to a change in directionality of these boundary conditions confers the transfer of information from this magnetic medium to the abstract language of computers.

### Property 1: orthogonal uniqueness of magnetic information

**Figure 2:**
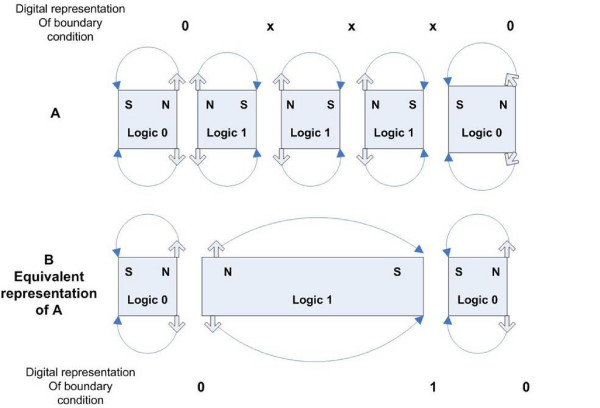
**Magnetic Boundary Condition**. In general, allowing a magnetic region to represent a logical "1" if magnetized N-S and a logical "0" if magnetized S-N results in a non orthogonal detection of flux transitions by the read head. Figure A shows that the intended pattern of bits "0 1 1 1 0" is not detected by the read head. Figure B shows the equivalent magnetic region layout which yields the detected bit pattern of "0 1 0."

It is imperative that data stored in any centralized system exhibit a level of integrity that enables them to be stored and retrieved without ambiguity. In their native state, magnetizing regions on a CHD disk, consisting of North-South (logical "1") or South-North (logical "0") dipoles, are not orthogonal. Figure 2A illustrates the problem for the binary bits contained in the sequence 0 1 1 1 0. Bits 2, 3 and 4 each representing a logical "1" do not exhibit a change in flux (polarity). This configuration is akin to placing 3 magnets in line with each other (such that north pole of the first contacts the south pole of the second); the effect is to create a single large magnet as opposed to maintaining three distinct ones (figure 2B). Consequently, there is no change in the regional boundary condition and therefore the read head cannot detect these bits. In order to remove this ambiguity, an encoding scheme is necessary to ensure that all combinations of logical binary sequences are unequivocally detectable with no chance of misreading or cross-talk. Schemes such as Frequency Modulation (FM), Modified Frequency Modulation (MFM) and Run Length Limited (RLL), all of which condition the magnetic data, ensure orthogonality is preserved [4]. The principle of orthogonality applies not only to logical data but also to Application Programming Interface (API) calls, macro invocations and language operations [6]. In terms of the CHD data, this information, whether represented as flux, voltage, optical bits or logical entity, is said to be orthogonal if each of its elements are unique, independent and have no cross talk attributes [6].

### Property 2: low level formatting of the computer hard drive (CHD)

Organization of the data structures on the CHD is critical for proper and reliable execution of computer programs. The CHD is organized such that data occupy physical space on the hard drive disk. This process is called low level formatting. Information stored on a hard disk is recorded in tracks, which can be visualized as a thin concentric circles placed on a disk. It would not be efficient for one track to serve as the smallest unit of information storage; programs may not need all the space provided by one complete track. In order to define more usable units of storage, sectors were developed to subdivide tracks into smaller, more manageable units. A sector subdivides tracks by introducing radially oriented discontinuities in them. This "pie slice" approach of dividing tracks into multiple sectors results in uneven sector lengths; this issue is addressed by the creation of zones composed of composite sectors to allow a more even distribution of storage space across the disk. Zoned bit recording organizes the sectors into zones based on their distance from the disk center. Each zone is assigned a number of sectors per track. Movement from the inner tracks occurs through sectors of arc length *l *with increasing circumference; each zone shows an increase in the number of sectors per track but a corresponding decrease in arc length *l*. This technique allows for more efficient use of the tracks on the perimeter of the disk and allows the disk to have greater storage capacity [4,5]. With this configuration the space made available to hold data has been organized in two-dimensional space to maximize the number of bits per storage unit. Further classifications of functioning and non-functioning sectors are identified and catalogued. This information is used by both the CHD controller and operating system so that data are not written to or read from these non-functioning sectors.

As part of the low-level formatting process, each sector has embedded information within its regions regarding its location, identification and data attributes. In the CHD, information that identifies every cylinder and track is called the track index. The track index tells the servo drive electronics where each track starts. In addition, information is provided in a region preceding every sector that guides the servo head to position itself precisely onto the requested track. This information is represented in a format called gray code and is written in a region called the wedge. In the ATA drive, the servo gray code is preceded by the track index. The function of this information will be discussed further in the hard disk controller section.

### Property 3: high level formatting of the computer hard drive (CHD)

Without a higher level formatting level of data organization working in conjunction with the operating system, data recovery from the CHD would be ambiguous. The operating system would not be able to locate specifically targeted packets of data reliably. Different operating systems use various ways to control and organize data for storage on media such as hard drives [4]. Operating systems need to manage the storage of information efficiently, accomplished through the development of partitions and other logical structures on the CHD. Partitioning the CHD disk is the act of defining areas on the disk that are operationally distinct, each containing the operating system(s) and files that the computer will use. Partitioning divides the hard disk into pieces called logical volumes. Given the number of files and directories that need to be organized for efficient storage and retrieval, these data objects are grouped according to a type of subject or classification paradigm. Files that share some common functionality, or need to share a common space for organizational reasons, are grouped into regions called volumes. These are logical structures used by an operating system to organize data stored on a medium using a particular file system. A single extended partition can contain one volume or many volumes of various sizes. Volumes can manifest themselves in what are called drives such as c: drive and d: drive (commonly used on PCs). These volumes are part of an organizational method used by a system called FAT (File Allocation Table) and are part of the high level formatting operation that is implemented through software contained in the disk operating system (DOS). Each partition or volume is then put through the high level formatting process by creating the FAT. Both functional sectors and zones, and "bad" sectors where data cannot be written, are identified, catalogued and stored in the FAT. Once this mapping has been implemented, further layers of organization fit the files and directories contained in the partitions and volumes to assigned sectors on the hard drive. Sectors are grouped into larger blocks called clusters, a process that occurs during the creation of the FAT. A cluster is now the smallest defined unit of disk space for storage of data [4]. For example, if a cluster is determined to contain 4 sectors which is equivalent to 2048 bytes (a byte contains 8 bits of data) and a file contains 2000 bytes, then the file is allocated one cluster.

Alternatively, a file containing 2100 bytes is allocated 2 clusters. With the cluster size defined and mapped to the partition, the FAT catalogs the identification and location of the clusters containing a given file, allowing the operating system to access the file when it is called for. The initial high-level formatting process organizes and maps files into contiguous clusters. However, as files are continuously written and deleted, new files may not reside contiguously on the CHD. Often they are mapped to different sectors on the disk, thereby causing the FAT to command the servo head to jump around the disk until it reads all the clusters that define the requested files. The process of distributing the clusters to different regions of the disk is called fragmentation. This can lead to decreased performance of the computer.

### Property 4: translation and access of the magnetic information via the hard drive controller

A hard drive controller is necessary in order to interpret API commands to locate and retrieve data on the disk, by steering the servo head to those precise locations. A precise servo control system allows the servo head to find the proper location specified by the FAT table. Once there, the servo head reads the data one bit at a time, which is converted to an electrical signal, decoded in hardware, filtered and loaded into a buffer. Finally, the data are transferred to the system bus via basic input/output system (BIOS) operations. In the CHD, instructions embedded in the hardware control magnetic pulse direction, amplification circuits, data manipulation (encoding/decoding/filtering), location of cylinder, track, sector or zone, precise servo head tracking of tracks, temporary buffer storage and data transfer. The hard drive controller (usually consisting of a dedicated CPU) responds to the previously described low-level formatted information held on the wedge area, specifically the track index and the grey code. This allows the servo drive to be positioned accurately onto the appropriate track allowing the servo head to read or write information to the disk precisely [4].

In the CHD, each sector has its beginning section reserved for management and control information [4,5]. Each sector contains a portion of its space reserved for information identifying attributes of each sector called the header region. The header contains identification information that is used by the CHD controller to identify each sector number and location relative to its track, and provides synchronization controls so the servo head knows where the data begin and end. It also provides a level of error checking code to ensure data integrity as well as indicating if the sector is defective or re-mapped. In modern drives, the header information is removed from the drive and stored in memory in a format map. This map informs the CHD controller where the sectors are relative to the servo data located in the wedge [5].

## Functional correlations between the CHD and the DHD

Having described four properties of the CHD that are essential for its function as an information storage and processing system we will now describe those aspects of the DHD that also fulfill these four properties. The emphasis in this section is not to attempt to draw one-to-one mappings between each component in the CHD and the DHD, but rather to describe the structure and machinery concerning the role of DNA in terms of the four functional properties of a centralized information-processing complex while noting specific instances where the implementation DHD diverges from the CHD.

### Correlation 1: orthogonality of the DNA genetic information

Biological systems also rely upon the property of orthogonality of information in order to minimize the chance of improper interpretation of the genetic language. Control regions, such as the promoters, insulator and enhancer sequences, and the codons contained in each gene, must be represented in a non-trivial and unambiguous manner. DNA nucleotides themselves have unambiguous attributes, contributing to the integrity of the DNA programmatic language. For genetic material, the boundary conditions required for orthogonality of information arise from the selective binding in nucleic acids, where adenine (A) pairs with thymine (T) and cytosine (C) pairs with guanine (G). The replacement of RNA uracil with thymine participates to orthogonalize the DNA molecule [7-9]. The DNA nucleotides A, C, T and G can be considered biological data units (Qbits) representing a base 4 system; in the context of the DNA molecule these nucleotides interact with various structural and functional molecules in their role of forming the "language" of genetic information. There is a functional equivalence between the orthogonality of magnetic representation of data on the CHD to the orthogonal representation of information in the form of Qbits on the DHD.

The generation of various types of RNA from the DNA code convert the coded information into a poly-functional format for use throughout the cell. The boundary conditions of the DNA and RNA code arise from integral biochemical properties of the nucleic acids that constrain their possible combinations. The interpretation of mRNA in the ribosome represents the "classic" role for RNA as a means of producing proteins; however, other functional RNAs, such as microRNAs (miRNA), large intergenic non-coding RNAs (lincRNAs) and small interfering RNAs (siRNA), serve as critical control elements in cellular information proccessing. The multiple roles of the RNAs suggests that RNA may serve as an information interpretation layer that is similar to the transfer of the magnetic flux encoding of the CHD into electrical voltage logic levels, which are then used ubiquitously in the computer logic circuitry.

### Correlation 2: Low level formatting of the DNA in eukaryotic cells

As discussed above, formatting of the data storage medium represents imposed organizational properties on the medium that facilitate the effective use of the stored information. As human DNA contains about 3 billion nucleotides constituting genes, regulatory sequences and other non-coding regions all residing in a one-dimensional sequence that is organized in 3-dimensional space, formatting of the DNA data structure is necessarily a far more complex issue than that seen in the CHD. This is particularly true because the "parts list" with which a cell is able to implement its data management is extremely constrained: nucleic acids, proteins and modifications thereof. Therefore, it is necessary to realize that the lines between "low level-," "high level-" formatting and translation/access functions may be blurred, since the molecular actors involved in effecting organizational properties may be the same. The poly-functional nature of RNA has already been alluded to; similarly, DNA, in what has previously been called its "junk" form, is being recognized as a critical actor in the organization and processing of cellular information [10,11]. This type of non-coding DNA, which constitutes approximately 94-96 percent of eukaryotic DNA, does not appear to participate in the "classic" Watson and Crick role of DNA as an information repository for protein synthesis; therefore the majority of human DNA appears to operate outside the traditional paradigm of the Central Dogma [12]. However, it is precisely because of the context-specificity of the roles of these molecular types that we believe it is important to parse the structure of the DHD complex into groups that may aid in defining classes of context, and lead to improved categorization of the various functions of the nucleic acids. Therefore, we first turn our attention to the physical structures that correlate to what we consider to be low level formatting, or physical organization of data structures, of the DHD.

**Figure 3:**
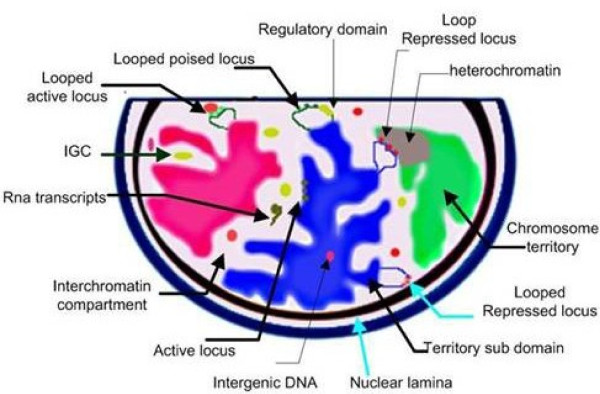
**DNA organization**. (redrawn from Kosak and Groudine, 2004). Architecture of DNA organization within the nucleus. Current view of how active genes are positioned in the nucleus and silenced genes are compartmentalized.

DNA is spatially organized within the nucleus [13]. DNA strands are compacted into chromatin and then subsequently organized into discrete chromatin territories (CT's) (see figure 3). The nucleus CT's are organized into regions of euchromatin and heterochromatin domains. Examination of the sub nuclear structure has shown genes collectively organize within their designated CT's. These regions are anchored to the sub-nuclear structure by a sequence of Matrix Attachment regions (MAR's) and Scaffold attachment regions (SAR's) [14-16]. Segments of repetitive DNA have been associated with the localization of these binding regions [17]. Closer examination has lead to the identification of intervening compartments distributed throughout the nucleus in the space between the CT's. These compartments have been suggested as a means of creating an interchromosome domain containing nuclear bodies needed for transcription splicing [18]. These peri-DNA structures demonstrate a level of spatial organization aimed at allocating transcribable domains of active and non-active genes inside the nucleus.

In interphase cells, evidence of a nuclear matrix consisting of a nuclear envelope and matrix-like nucleoskeleton shows both loops and MAR/SAR attachments connecting the DNA to the nuclear structure [14,15]. The nuclear matrix is composed of ribonucleoproteins such as lamins found ubiquitously throughout the nucleus. Lamins are present in the nuclei of all eukaryotic cells and form a rim like structure on the inner layer of the nuclear membrane, but also a deep intranuclear tubules forming a veil like network. The nuclear lamin interacts directly with DNA in chromatin [19]. This 3 dimensional network forms the Nuclear Attachment Substrate (NAS) which is a physical structure analogous to the disk and track layout of the CHD. The DNA organized within the CT's is structurally anchored and may be spatially organized within the nucleus in terms of partitions and volumes (discussed in high level formatting section). Recent observations suggest that transcriptionally non permissive regions of CT's are organized near the nuclear membrane periphery while transcriptionally permissive genes are located deep into the nucleus [20]. Insulator bodies can co-localize in large foci to the sub nuclear structure forming clusters of genes. It is unclear as to the mechanism that defines the location of the MARS/SARs/insulator sites, however it is clear that the functional characteristic of the nuclear attachment substrate is analogous to the spatial layout of tracks adhered to the disk of the CHD. In this case the DNA polynucleotide molecule is considered to be a super track. The "track" of DNA is composed of alternating molecules of sugar ribose and phosphate forming the structure to hold the data ie, bases of Qbits. This is directly analogous to the tracks on the CHD that provides the boundary constraining the magnetic bits to contiguously and linearly align, as the sugar ribose phosphate moiety acts as the boundary that aligns the Qbits within the structure of the molecule forming nucleotides. However, it should be noted that this does not mean that the data (Qbits) will be used in a linear contiguous fashion, as will be seen to be evident through fragmentation and alternate splicing. This description is consistent with our definition of low level formatting.

The main function of low level formatting is to organize the storage space in the DNA/sub-nuclear hard drive coherently via its sub-nuclear structure. This allows the nuclear machinery to operate upon the CTs in the euchromatin for such tasks as copying, splicing and other regulatory functions. However, a higher-level structural organization is present that facilitates the ability of the cellular machinery to accomplish these tasks, and is manifested in the higher order chromatin domains. The DNA hard drive paradigm can now be assembled using two principles, physical structure (low level format) and software abstraction (organizational management). The second principle involves dividing the genome into logical pieces called partitions and further organizing the data into volumes and clusters using a process called high level formatting. Table 1 summarizes the comparison between the CHD and DHD relative to the low level formatting process

**Table 1:** Low Level Formatting Comparison

Computer Hard Drive	DNA Hard Drive
Track	The entire DNA strand as one large super track defined by the sugar ribose phosphate back bone. This super strand exhibits connectivity to the Nuclear Attachment Substrate (NAS) consisting of lamin networks.

Sector	That length of track that encompasses the gene/genes, promoter/Basil Transcription Complex consensus sequences and other distal sites bounded by insulators attached to the nuclear lamin.

Servo wedge info	Promoter regions.

Synchronization header	Basil Transcription Complex consensus sequences enabling factors such as DPE/Inr's that sync RNA Pol II to the initiation start site.

### Correlation 3: high level formatting of the DNA: Posting a Biological File Allocation Table

In the CHD, high level formatting begins with partitioning the hard disk into discrete isolated regions. Partitioning in the CHD accomplishes the following purposes: 1) This allows grouping of related and similar data and operations together to improve efficiency of utilization. This efficiency is both mechanical, reducing the distance the CHD read-head needs to traverse in order to read related data/instructions, and operational, as smaller cluster sizes reduce "slack" (the potential unused space within a cluster) thereby increasing performance and efficiently utilizing disk space; 2) Isolation of regions facilitates the restriction and recovery of corrupted files and data. If one partition is corrupted, isolation protects the other file systems from being affected, thereby increasing the chance that some of the drive's data may still be salvageable, and avoiding total system failure; 3) Partitioning allows a single CHD to utilize multiple operating systems. In our model, the DHD can be considered to be partitioned into chromosomes. These form discrete physical entities of genetic material, and are the functional units that serve as the vectors for the transmission of genetic material from cell generation to cell generation. As such, there are evolutionary implications of this type of organization related to the robustness associated with modular information storage units, specifically in terms of the relation between selection forces, the units being selected and the maintenance of survivable functionality in the carrier phenotype (this will be discussed in more detail below). To some degree, the presence of multiple chromosomes in eukaryotic cells can be considered to represent multiple "drives" of the DHD, these drives further divided into extended partitions of euchromatin (denoting protein coding DNA) and heterochromatin (representing control/suppression roles for non-coding DNA to be discussed further below). However, the isolation of regions resulting from "partitioning" of the DHD is not a rigid as in the CHD. Regulatory pathways and metabolic modules may require information that crosses chromosomes, as information for a process initiated on one chromosome can be accessed and acquired from another. Therefore, the functional/logical organization of the DHD calls for further refinement beyond the organization of the CHD.

In a CHD, volumes are logical structures representing the top level (i.e. most inclusive) of file organization. In the DHD analogy, data volumes can be characterized by the content of heterochromatin and euchromatin regions imposed in part by MAR/SAR attachment points and the histone code. There is considerable evidence that the nuclear architecture is closely related to genome function and gene expression [21]. The consequences of this spatial organization are evident during cellular differentiation, when alteration in the sub-nuclear structure enables some types of gene expression while silencing others. As genes are silenced, the extent of chromatin condensation is seen to increase. Recent studies suggest silent chromatin may influence nuclear organization [22,23]. It is also noted that the distribution and amounts of condensed chromatin are similar in differentiated cells of the same lineage but vary among the nuclei of different cells [24]. The extended partitioning of the CTs are manifested by their compartmentalization within the nucleus. An additional degree of functionality is present in the extended partitions within the CTs, enabling a transcription state of active or in-active chromatin domains. Chromatin domains are in this sense dynamic logical structures with respect to gene expression. The action of the histone code and cell control circuitry dynamically alters the compartmentalization of active and non active domains along the DNA as a function of epigenetic expression. Structural organization within the nucleus exhibits a dynamic quasi - steady state (as opposed to a purely steady state configuration). This organization changes in time and represents a dynamic topological organization of genes and their control codes within the organizational structure of the nucleus. The histone code and its control mechanisms are considered to be part of the high level formatting process, responsible for the creation of both the extended partitions and their logical transcriptional state (on/off).

**Figure 4:**
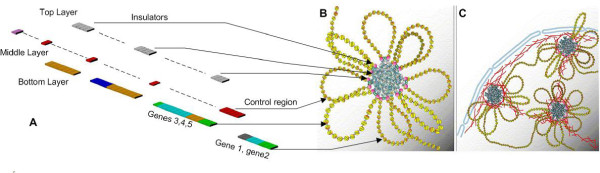
**Organized cluster mapping of DNA to Nucleus**. Mapping of DNA strand into DNA Hard Drive: A) shows the DNA strand decomposed into its information structure. The top layer (gray) contain the strategic placement of insulators, the middle layer contains the regulatory control regions (red) that controls the copy process of the genes and the bottom layer contains the genes organized into a form that allows co-expression. B) Shows the mapping of the insulators to the nuclear lamin substrate to form insulator clusters. These cluster are placed such that they structurally partition the genes into organized clusters. The regulatory control regions (red) now become specific to the rosette pattern formed from the insulator clusters. This results in a rosette pattern of genes and their control regions. C) Shows the placement of the rosette patterns to the nuclear lamin substrate within the nucleus thus creating the DNA hard drive. The red lines indicate the lamin. Pictures B and C from Maya, Corces, Capelson and Victor, "Biology of the cell" with permission. Available online 09 September 2004.

**Figure 5:**
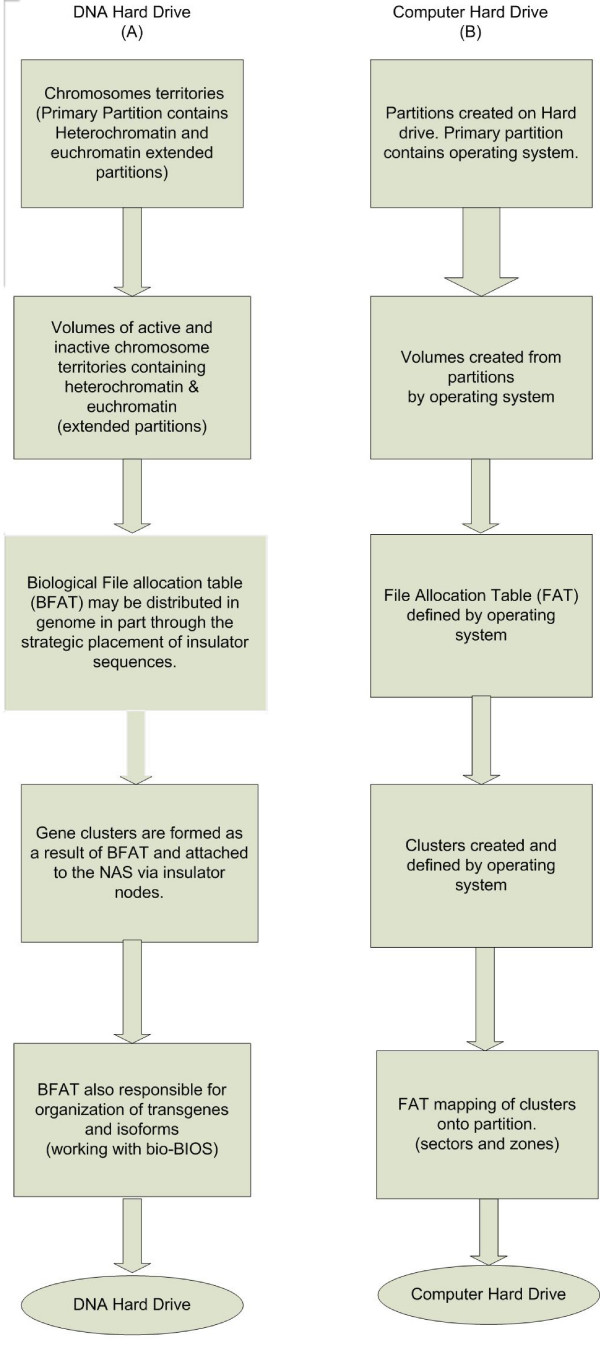
**Flow chart comparison of high level formatting of DNA and CHD**. Formatting models of both the DNA Hard Drive and the Computer Hard Drive. Figure A shows the path for high level formatting of the DNA molecule. Starting with the physical organization of the chromosomes into specific territories which then results in high level formatting layered on the DNA molecule itself and finally implemented onto the sub-nuclear lamin in the form of rosette patterns of gene clusters. Figure B consists of the computer hard drive illustrating high level formatting processes. Notice the similarities between the two models which show a degree of functional equivalence.

The CHD is further organized through the creation of data organization units physically allocated over one or several disks called clusters. Recall that CHD clusters are the smallest organizational unit of data storage transposed to the disk; similarly, biological data clusters are the smallest working units of transcribable genes. If genes are defined as individual data files, these clusters of genes can be seen as clusters of files located within the partition and volumes defined by CTs. The cluster size is defined by the placement of insulator consensus sequences in the genome and consequently placed on the DHD by attaching the insulator attachment points to the proper nodal connections on the nuclear lamina. The genome in our model can be thought of as a polyfunctional assemblage of nucleotides organized into layers of insulator consensus sequences, regulatory regions and codons (Letter A in figure 4). The non-random linear arrangement of gene clusters [19,25] and the placement of insulator consensus sequences on the DNA result in a highly ordered structure and extended partitioning of the sub-nuclear lamina. This suggests a hierarchal organization of information leading to transcription and cellular differentiation. One type of cluster may be made up of arrangements of genes that co-locate to a common node on the sub-nuclear substrate through the nodal attachment of insulator sites, sometimes forming a rosette pattern of chromatin loops (Letter B in figure 4). The reference system for identifying and describing the insulator effect of these higher-level chromatin domains is the Drosophila genome. Data from Drosophila suggest that static domains form as the result of additional compartmentalization of chromatin that can function as insulators, which can have a further effect on gene expression [25-27]. Loop formation requires an intact nuclear matrix [28]. The interaction between multiple insulator sites coming together at specific nuclear locations (Letter C in figure 4) is in part related to the distribution of insulator consensus sequences resulting in the formation of chromatin rosette structures [16,19]. This evidence supports the argument that the insulator bodies act as attachment nodes for data (gene clusters or active transcriptional domains) to specific locations within the nucleus in a manner that parallels the function of placing binary data into clusters in a formatted computer hard drive. A model of the high level formatting process is shown in figure 5.

Alternatively, clusters may also be formed by physically separated sequences that are co-expressed and brought together by higher-order control mechanisms (to be discussed in the next section on information translation and access). Note that this latter case is similar what happens over time on a CHD as new data is cycled through the system, as previously contiguous clusters become distributed throughout the CHD in a process called fragmentation. DNA fragmentation occurs when unlinked exons of a given gene are distributed throughout the genome analogous to clusters of a given file in the CHD, allocated to non contiguous sectors. In order for the system to continue functioning over time, a mechanism must be present that allows the acquisition and re-ordering of these distributed data objects. In the CHD, clusters for a given file are mapped by the FAT which directs the read head to the appropriate track and sector where it is read and sequentially placed into the read buffer until all of its clusters are in the proper order reconstructing the original file. Extending this analogy to cells would imply a biological map analogous to a FAT that defines where these genes are located, what we term a Biological File Allocation Table (BFAT). What constitutes the BFAT? In a CHD, the FAT is imposed during installation of the operating system and is stored on the disk; in the DHD there is no external imposition of an equivalent organizational schema. Rather, this information is, in part, embedded somewhere in the cells genetic code, leading to a recursive data-control relationship. While we do not know whether such an equivalent BFAT exists, the models we are building strongly suggest it. The operation of the genome, in particular the insulator node clustering, appears to support the implementation of a BFAT. We propose that reading fragmented genes in the DHD occurs through the process of trans-splicing and actions of the RNA-incuded silencing complex (RISC). Our model predicts that the fragmented exons of a given gene must be mapped by the BFAT which is then acted upon by the cells regulatory circuitry to copy biological sectors, each to its own pre mRNA buffer. The BFAT then mediates the spliceosome to collect the appropriate exons from the multiple pre mRNA's, multiplexing them sequentially to reconstruct the requested gene transcript.

There is also recent evidence of an even higher level of organization amongst the clusters of the DHD. Within a single gene, non-continuous formations of exons and introns have been found to generate more than one protein product via the expression of alternative spliced mRNA isoforms [29,30]. These selective combinations of exons suggest the existence of multiple temporal mappings. Multiple temporal mappings means that for a given gene consisting of x introns and y exons, there a multitude of combinations of the given exons and introns that when put together into one contiguous order, represents an alternative form of the primary gene (isoform). These mappings are controlled by signal transduction pathways acting and nuclear transcription factors. As external and/or internal conditions vary, cells can call for one of these mappings at different times of its life cycle, hence the term temporal. Since this would require multiple mappings, BFAT is presumed as a candidate to store these mappings such that the splicosome can rearrange the exons accordingly. Such mappings fit within the definition of a distributed BFAT. These mappings could potentially be part of a localized operating system for a specific type of differentiated cell, and are executed by the cell's editing hardware (i.e. the spliceosome complex). The ability of the spliceosome to re-sequence exons for a given mRNA requires close coordination of Bio-BIOS and the DHD controller (discussed later) along with BFAT for proper isoform construction. The combinational sequence of exons in one gene sector potentially contains more information carrying capacity than that of one single contiguous sequence of exons nominally defined within a gene. The DHD controller orchestrates the actions of the spliceosome through the coordination of RNA regulatory and splicing factors, effectively multiplexing the relevant exons into a mature mRNA where it is packaged and serially sent to the ribosomes. This represents a higher level of organization than the conventional removal of introns from a typical pre-mRNA. Isoform mappings fit well within the definition of the BFAT.

It is worth noting that in terms of cellular behavior identifying when the "start point" occurs becomes extremely vague. However, in drawing our analogy to equivalent stages in CHD data management it is reasonable to focus on stem cell differentiation as being similar to the formating and partitioning stage for the CHD in preparation for loading its operating system. In this circumstance, the distinction between low-level and high-level formatting becomes blurred. As stem cells differentiate and mature their DNA can dynamically alter its organizational configuration within the nucleus by restructuring its euchromatin and heterochromatin compartments via histone modifications and reconfiguration to the sub-nuclear lamina. To a certain degree, stem cell differentiation can be considered dynamic low level formatting as this process results in regions that may determine the architecture of active and inactive gene regions depending on the trajectory of cellular differentiation. This degree of control and adaptability is much more sophisticated than the technology employed in hard drives, where the sectors along with their state of activation remain unchanged after low level formatting. Key processes involved in the regulation of the euchromatin and heterochromatin compartments are increasingly being linked to non-coding segments of RNA. In addition to the role of RNA interference (RNAi) in silencing segments of genetic material, non-coding RNA has been implicated in the formation of heterochromatin and the development of higher level gene-oriented structures such as centromeres and telomeres [31]. Additionally, lincRNAs, which appear to be the products of repetitive DNA, have been shown to play a role in guiding chromatin-modifying complexes [32]. It is becoming increasingly clear that non-coding RNAs play a vital role in the epigenetic regulation of cellular information processing, particularly in the construction and configuration of genetic data structures at a supra-transcript level. When cells begin to differentiate, it is logical to assume that multiple genes that perform functions relative to the cell type should be sequestered to regions of the nuclear lamina that will facilitate in their transcription. This may be accomplished by re-grouping insulator nodes to the sub-nuclear structure along with MARs and SARs and implementing unique histone logic programs. This represents a point of departure from the CHD analogy, and would be equivalent to the tracks and sectors re-configuring its connectivity to the disc. Likewise, multi-gene compartmentalization in heterochromatin may need to be arranged so that they may be silenced. An underlying linear order of genes arranged along the chromosome accommodates the coordinated regulation of transcriptomes. For example, IgH and B-globin loci share common genomic positions that are regulated in specific cell types [25]. These linear arrangements of genes coincide with nuclear localization patterns that facilitate their state of activity. Even though these two gene arrays are the result of duplication events, the co-regulated homology may yet be organized in linear clusters throughout the genome [25]. This may represent coherence between a co-regulated linear arrangement of genes on the DNA and their physical placement in the nucleus. It is proposed that the linear arrangement of gene clustering into transgenomes meets the defined criteria of high-level formatting required of centralized information processing systems. Table 2 summarizes the comparison between the high-level formatting of the CHD and that of the DHD.

**Table 2:** High Level Formatting Comparison

Computer Hard Drive	DNA Hard Drive
Partitions	Physical compartmentalization of chromosomes and chromosome territories, extending to heterochromatin and euchromatin regions. These are imposed in part by MAR's/SAR's attachment points, histone code, repetitive DNA and other non coding RNA's.

Volumes	Logical space allocated to chromosome territories including heterochromatin and euchromatin regions.

Clusters	Gene sectors defined by the clustering of insulators nodes.

File Access Table (FAT)	The implementation of the biological equivalent FAT (BFAT) is manifested by the strategic placement of insulators and enhancer consensus sequences superimposed on the genome. This results in particular insulator clustering in conjunction with inter/intra cellular communication with the epigenetic system. Congruent with the architectural layering of insulator consensus sequences distributed within the genome, BFAT may also be realized within the wetware circuitry of transduction signaling pathways representative of a form of cell firmware.

### Correlation 4: translation and access of biological information via the DNA transcription machinery

One of the limitations of the Central Dogma (and, for that matter, the abstract description of a digital computer as a Von Neumann Machine), is that the abstract representation suggests a linear process: one sequence of DNA leads to one mRNA leads to one protein. Clearly, in terms of the cell, this is not the case. The cell manages multiple processes concurrently rather than as a single threaded sequence. However, despite its multi-threaded computing capacity, a cell retains a single set of chromosomes residing in a centralized position, both spatially and organizationally. Therefore, to draw our analogy out more completely the cell is viewed as a complete computational machine in terms that are akin to a multi-core computer cluster, where there is a centralized memory and instruction set, yet computational tasks are distributed among distinct processing elements. We will return to the concept of the cell as a multi-core computing device in a following section, but in order to finalize the determination of equivalence between the CHD and the DHD we will attempt to describe the biological analog to a single thread of information processing.

For a cell to utilize the information contained in its chromosome requires that the intra-nuclear information encoded on DNA be converted into a form for use throughout the cell. As alluded to above, the various types of RNA serve as intermediaries in the translation, access and control of the information encoded on the DNA. mRNA is the intermediary data format for protein synthesis, and for purposes of comparison to the read head of the CHD, will be the focus of discussion in this section. While non-coding RNAs play a critical role in genetic information processing, their post-transcriptional role in modulating how the genetic instruction set is implemented more closely approximate the functions of the CHD's instruction set and operating system; a posited role and analogy in this context will be presented in a later section. This is yet another example of the complex challenge of characterizing cellular information processing arising from biology's use of poly-functional components in the name of "economy."

The conversion of DNA information into mRNA information requires locating the appropriate reading frame, identifying the initiation start site, and reliable copying of the data file. Each gene is associated with a code sequence called the promoter region that contains information that initializes the biological equivalent of a "read head" at the proper transcription initiation site of each gene. In the eukaryotic DHD paradigm, we consider the RNA polymerase II complex (RNA Pol II) as performing functions analogous to the CHD read head. The promoter region acts as the foundational footing for the assembly of the necessary molecular components (transcription factors and cis-regulatory elements) into the Basal Transcription Complex (BTC), which ultimately aligns RNA Pol II to the transcription initiation site. The BTC aligns and attaches to the proper TATA sequence forming the reference base that will participate in properly aligning the biological read head. The spacing of the nucleotides between the TATA box and the transcriptional start site is critical in determining the proper open reading frame for the start of transcription [33]. Misalignment of the RNA Pol II would cause a reading frame error. Attachment of RNA Pol II to the appropriate transcription initiation site is facilitated by the correct alignment of Transcription Factor II B (TFIIB). TFIIB is a multifunctional molecular complex, a signal receptor that responds to gene activation molecules. RNA Pol II attaches to the TFIIB complex, resulting in conformational changes that enable RNA Pol II to target the initiation start site accurately [33]. In this sense, the Basal Transcription Complex is functionally equivalent to the servo head, wedge gray code and synchronization portion of the header control sections of the CHD.

The interactions of activators with their corresponding core promoters take on the form of dynamic changes in both the chromatin and the assembly of general transcription factors, such as the conformational change of the RNA Pol II complex [33]. This is vastly different from the control circuitry of the CHD. In the CHD, the routines of locating and extracting data using the read process are both mechanically determined and controlled by pre-defined logic. The combination of core promoters and activators permits gene regulatory activity that far exceeds the level of control in the CHD controller circuitry. While the DHD can adapt to changes in the cell's environment, the CHD is much more restricted. The header portions of the genetic files offer a higher degree of freedom in terms of active control then the preset headers of the CHD. The fashion in which RNA Pol II travels as it reads the transcribed gene is quite different as well. In the CHD, the disk containing the magnetized bits of data rotates at a high speed beneath the servo head. As the disk rotates, a new bit (flux boundary) travels across the receiving boundary area of the servo head and the data are read. In the DHD, the DNA can be considered to remain relatively stationary while the RNA Pol II is in motion. Furthermore there is a leading unwinding of the nucleosomes that allows the RNA Pol II to read each nucleotide effectively. A mechanism called the RSC chromatin-remodeling complex, a protein machine, unwinds the wrapped DNA strands. The RSC effectively holds the individual nucleosome and creates a propagating bulge in the histones that exposes the DNA strand for transcription [34]. This is all done in relation to the sectors and clusters embodied within the sub-nuclear structure. After the RNA Pol II copies a nucleotide, the complex advances and the other machinery collects the histones and repacks the DNA. This process is much more complicated than the CHD, but is consistent with the CHD in that there must be relative motion between the data storage medium and the mechanism that reads it. Table 3 shows the mapping between the corresponding read head functions of the CHD and DHD controller functions. It is interesting to note that the control signal to execute the transcription action is typically based upon interactions with the distal promoters and enhancers. Enhancers can be thousands of base pairs away from their associated core promoters. Enhancer-bound factors can literally take part in bending the DNA track such that they physically communicate with their core promoters. This can trigger the transcription of an active gene. This bending of the DNA is a three-dimensional structural change that has no counterpart in the CHD. Because the promoter regions are coupled to their corresponding gene and/or gene cluster, the protein-centric view of a gene is being reconsidered to include in its definition the regulatory and transcriptional regions and other non-transcriptional sequence regions [35]. Using this definition, it is proposed that the gene, along with its promoter/Basal Transcription Complex, is consistent with the physical layout of the sectors with headers in the CHD and is functionally equivalent to the sectors in the DHD. In both cases it is up to the controller to identify the requested regions, check and confirm that they are enabled for copying.

**Table 3:** Comparison of CHD controller functions to DHD controller functions

Computer Hard Drive	DNA Hard Drive
Track Index	Unknown (possibly enhancers)

Servo Wedge alignment information	Gene promoter regions such as the TATA, Inr and DPE sites and transcription factors accurately aligning the Basal Transcription complex to the gene/sector.

Read Head (Servo Head)	RNA Polymerase II (eukaryote)

Synchronization of servo head to read data (located in header)	Synchronization of RNA Polymerase II to initiation start site by proper alignment to the promoter site by transcription factors. This allows conformational alignment of RNA Polymerase II to transcription start site (part of biological header complex)

Identification of active sectors (header)	Implementation of the histone code. In addition, regulatory elements as well as other possible non coding RNA's influence RNA Polymerase II. (Part of biological header)

Read/Write data acquisition is through rotation of disk platter relative to servo head.	RSC chromatin remolding Complex unwinding DNA double helix in conjunction with RNA Polymerase II (read head) moving step wise along the DNA strand.

Data buffer and sector editing	Sub sector editing/multiplexing of both exons and sectors implemented by splicosomes and wetware circuitry of the DHD controller. This machinery reconstructs the requesting genomic information and its derivatives (including transgenes and isoforms) resulting in a mature mRNA residing in the buffer region of the nucleus.

Bios	Bio-BIOS consisting of transduction circuitry resulting in pathways responsible for translating cellular requests into the RNA regulatory language of the DHD.

## Discussion: Advancing from discrepancies in the comparison between the DHD and CHD

Despite their similarities in functional properties as centralized information storage and processing complexes, there are significant areas where the analogies break down. These arise largely because the specifications of the CHD are imposed from an external source and it is constructed using external resources and machinery, while the DHD must contain and implement the means for its own construction and upkeep. This "autopoetic" capacity necessarily adds layers of complexity to the structure and function of the DHD. However, by recognizing the components necessary for centralized information storage and processing, the nature of the DHD's expansions on the CHD can be more specifically characterized and linked to known biological components and their functions.

### "Economy" in the cell: the varied roles of DNA and RNA

One of the most immediately evident discrepancies in any comparison between a digital computer and a biological cell is the fact that digital computers are engineered systems and cells are evolved systems. There is an implicit imposition of structure and design in a digital computer, and while there is always going to be a tendency towards efficiency in the design of computers, the evolutionary paradigm places component constraints front and center. This is particularly notable in the fact that the cellular computer has a finite number of pieces with which to work; it is starting with a constrained parts list, and as a result nucleic acids need to be viewed as polyfunctional objects. This is particularly evident in the recognition of the role of junk DNA in data formatting and process control [10] and the multiple functions of non-coding RNA [31]. A comprehensive description of these areas of research is beyond the scope of this manuscript, however, the recognized polyfunctional nature of DNA and RNA does suggest the need for a considerable expansion of the traditional view of nucleic acids. There are significant implications to this viewpoint, because now it is the context of the nucleic acid that determines its function and classical molecular characterization based on base sequences and molecular weight are insufficient representations of the informational structure of the DHD. The need for a more expansive view of nucleic acids is evident in our comparison between the DHD and the CHD; the functional characteristics needed by a centralized information processing system require multiple levels of control. Given a limited "parts list," it is inevitable that given the constraints of "economy" in the evolutionary process we find basic components being put to multiple uses, similarly seen in the economizing of engineered designs. Furthermore, the principle of "economy" of components also suggests that the diversity necessary for natural selection to work must reside in the organization of these components. The initial implication of this situation leads to the traditional concept of the unit of selection acted upon by natural selection: organizational patterns of genetic information tied to a particular function. But the expansion of the functional roles of nucleic acids beyond the coding of identified proteins adds additional dimensions to the aspects of the cell's information processing system, and expands the description of the functional unit that may be selected for from one generation to the next. It is possible that the segments of repetitive DNA and the non-coding RNAs represent, if not exact sequences, then repositories of critical mechanisms necessary for the maintenance of cellular function. Remembering that natural selection acts upon the phenomenological consequences of a particular mechanistic structure, understanding and recognizing the classes of essential functions required for cellular activity, such as information storage and processing, is a critical step towards what constitutes a "selectable" unit. Furthermore, identification of the "selectable" unit is challenging due to the simultaneous multithreading processes contained in the cells information system. We believe that our DHD to CHD comparison accomplishes this for a vital sub-group of these essential functions: those associated with the core centralized information storage and retrieval actions delineated in the four functional properties described above. While we believe that we have established a mapping between the CHD and the DHD with respect to the four essential properties of centralized information storage and processing, expanding the CHD/DHD analogy requires understanding and defining the additional information processing activities necessary in order to maintain a cell, for just as a digital computer is an expansion beyond the abstract description of a Von Neumann machine, the cell is an expanded, concrete expansion of the traditional Watson and Crick Central Dogma into a biological machine. Therefore, it is natural to look to the non-traditional roles of the nucleic acids to provide insight into the "missing" computing pieces of the cell. With this in mind, the following sections present examples where nucleic acids are found in non-traditional roles in the course of expanding upon our comparison between computers and cells.

### Biological BIOS (Bio-BIOS): firmware for the cell

When computers are turned on, a sequence of non volatile instructions cascade from that initial time point, resulting in the dynamic information processing device we use [4]. Cells, however, do not have a readily identifiable start point ; even new daughter cells arise with their information storage and processing machinery already engaged. This makes it difficult to draw analogies between the basic setup processes in the CHD and putative initiation sequences in the DHD. However, identifying the function served by these processes in the CHD allows the molecular components that serve the equivalent function in the DHD to be sought. The search for a form of Bio-BIOS is just such a case.

In general, a sufficient condition for information in system A to communicate with system B requires a mapping to define the interface between the two systems. The mappings may consist of a set of instructions or circuits that act as a translator between the two domains. In computers, this instruction set is called the basic input/output system, or BIOS, and has been described as firmware to characterize its place and role between software and hardware. In a computer the power up process loads initialization instructions residing in read only memory (ROM) and proceeds to load BIOS drivers so that the operating system (once loaded) can communicate with its peripherals, including the hard drive. System calls to read data from the CHD are processed by the operating system (OS) and referenced to FAT before being sent to the CHD. The operating system does not know how to directly communicate with the CHD (or any other peripheral) and must translate its generic request via drivers or BIOS. BIOS translates the OS request for data into the language of the CHD. The translated request from BIOS are interpreted by the CHD's internal controller which sends the appropriate signals to read/write head and editing buffers. Using this model and applying it to the cell, our analogy suggests an equivalent biological BIOS exists.

We suggest that a form of biological BIOS resides outside the nucleus and is responsible for translating cellular requests into the RNA regulatory language of the DHD. As an example, regulation of the PKC gene through alternative gene splicing stimulated by insulin produces the BII isoform of this gene. In this instance, Bio-BIOS initiated by a hormone signal cascades into a series of metabolic reactions through transduction circuits located in the cytosol. This produces the protein serine-threonine Kinase which travels into the nucleus [30]. Once there, RNA elements act upon it, leading to the phosphorylation of Srp40. Srp40 then binds to intronic elements of the PKC mRNA leading to the inclusion of the BII exon [30]. This signal transduction pathway in the cytosol is posited to be functionally equivalent to BIOS.

The DHD Controller is defined to encompass RNA's and protein factors that govern the operation of the DHD. The editing machinery is composed of spliceosomes and all exonic and intronic enhancers that enable cis splicing and all its derivatives. It is posited that the regulation and control of the nucleus has the characteristic functionality of a controller to the DNA hard drive that meets and exceeds the criteria of an equivalent controller function residing in the CHD. It is further posited that each cell type or phenotype has its own individual form of BIOS that translates intra or inter cellular requests to the DHD. Examples include regulation of the BK STREV exon by neuronal activity, inclusion of the alternative exon of the ICH-1 gene by Ischemia and regulation of alternative exon v5 of the CD44 gene after T-cell stimulation [30]. In each of these cases, requests from the cell have to be mediated such that the proper interpretation is sent to the nucleus for execution by the DHD. Congruent with the architectural layering of insulators, BFAT may interact with the wetware circuitry of Bio-BIOS completing the communication pathways between cellular processes and the DHD.

### Biological operating system (BioOS): control software for the cell

**Figure 6:**
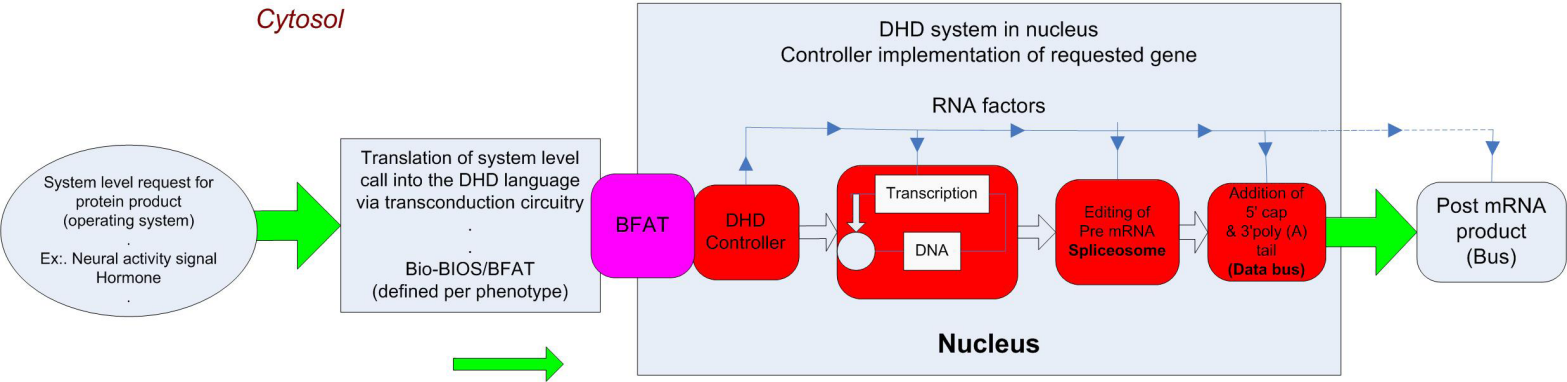
**System diagram representing information request/process**. The cell produces a system level call for a protein product. The request is processed by the operating system (BioOS) and is translated by Bio-BIOS (transduction circuitry firmware) into the DHD language (which may be a function of BFAT). Translated signal from Bio-BIOS is sent to the nucleus. Once in the nucleus, RNA/protein circuitry defined as the DHD controller assembles the transcription process. Transcription of the DNA produces the pre mRNA which becomes a temporary buffer. Final editing is accomplished through the spliceosome as implicitly defined by BFAT for the proper RNA copy of the requested gene including its derivatives via alternative splicing. Finally, a 5' cap and 3' poly (A) tail is added to the edited mRNA enabling it as a serial bus structure. Additional control effort are leveraged against the post mRNA regulating the protein production process.

We propose a general description of the characteristics of a DNA operating system that operates on top of the centralized information storage complex within the nucleus, and provides the specification of the execution instruction set by which cellular information is utilized by the cell. This system is responsible for executing the organization of the CTs through the functions of the histone code, essentially providing a dynamic mechanism for modifying the low-level formatting of the DHD where repetitive DNA likely plays a critical role in terms of specifying partitions/volumes within the CTs and guiding reorganization of the genome when needed [10]. The BioOS enables the cell to communicate with the DHD and other epigenetic functions. Non-coding RNA represents the putative candidate for the components of the BioOS. As noted above RNA serves a series of functions aside from its classical transcriptional/translational role, including those of recruiting entity, scaffolding factor, and sequence-specificity determinant involved in targeting histone modifications [31,36,37]. RNA interference (RNAi), acting through miRNAs and siRNAs, has been shown to suppress mRNA and represent a post-transcriptional mechanism for modulating gene activity. siRNAs are integrated into RSC complexes to target exactly matching segments of mRNA, interfering with mRNA activity and therefore protein production. Furthermore, as described above, non-coding RNAs play a critical role in controlling the configuration of the chromatin domains, and therefore the access to information stored on the DNA. RNAs may also influence the genome organization through instruction templates passed from parent to offspring [38]. All these functions suggest a critical role for RNA in the control of cellular information processing. Figure 6 shows a system diagram representing the information request process. The implications of this are significant from a potential reverse-engineering standpoint, as RNA appears to have a role as media for both representing and implementing information; functions that are currently distinct in engineering computer systems.

### Multi core cells? Non-uniform memory architecture and distributed computation within the cell

As noted above in Correlation 4, the cell processes multiple computational tasks in a concurrent fashion. It is as if there are multiple servo heads reading various aspects of the DHD at the same time, each data stream leading outwards to a distinct computing unit. One way of looking at a cell, then, is as a system with centralized memory with multi-access capability leading to distinct computing units. This requires, a further degree of organization that goes beyond a mere description of a read-write capability. Rather, there must exist within a cell a mechanism by which multiple threads of information processing are maintained. We hypothesize that this role is played by the BFAT, which is distributed throughout the genome in a manner similar to the non-uniform memory architecture (NUMA) used in distributed memory design [39,40]. A distributed BFAT might be localized to a sector or a cluster capable of interacting with the RNA machinery, responsive to a cellular request for specific proteins. It would be responsible for the hierarchical organization of a seemingly fragmented genome, and form the control system that splices these genes together to enable the protein products to be made. In this model of the BFAT, its organizational structure is superimposed on the DNA genome through the strategic placement of insulators and genes [25,41] (see figure 4). Thus the BFAT table is in part, built into the layout of the DNA molecule.

**Figure 7:**
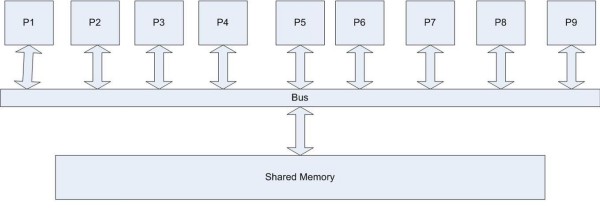
**Shared Memory**. Processors P1 through P9 all share the same memory as they each wait their turn in the queue.

**Figure 8:**
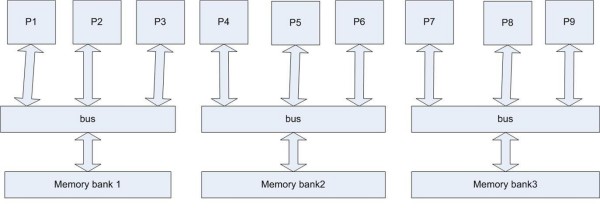
**NUMA**. Three memory banks, or nodes are designated for each group of three processors. This allows nearly equal access times for any processor within the node. This assumes that the processors within each node are relatively the same distance from the node.

When multiprocessors are used, each processor can access its own local memory, i.e. standard memory such as random access memory (RAM), much more quickly than non-local (shared) memory. NUMA architecture was designed to surpass scalability limits imposed on Symmetric Multi-Processing (SMP) also known as Shared Memory Parallel, architecture found in computer systems that have relatively few CPU's [39]. SMP allows all processors to have access to the same memory bus creating wait states for CPU's competing for access to the bus. This is because only one processor at a time can access the bus (see figure 7). NUMA reduces the wait states by re-grouping the numbers of CPU's to discrete memory banks connected by its own memory bus, collectively called a node (see figure 8). A general description of a node is considered to be a block of memory, CPU's, input/output (I/O), etc, physically on the same bus as memory [42]. NUMA may be scaled to handle hundreds of CPU's, creating clusters of nodes allowing faster access to local memory than in shared memory architecture. With respect to the DHD, its data that defines the proteins and RNA's used in the construction and maintenance of the cell is contained in the memory pages of the genome. Data needed to build protein/RNA machines are contained in local memory regions defined as insulator cluster nodes, perhaps defined by regions of repetitive DNA and leading to the rosette patterns of chromatin loops located within the interchromatin space. Such nodes as defined by BFAT are similar to NUMA nodes. Further information not found in the local memory may be accessed in a different nodal cluster (remote memory). The cell in our analogy is considered to be an aggregate of CPU-like processes, each requesting data from the genome in response to environmental or other internal/external processes. Setting up the DHD as described above allows each cellular CPU-like process to access information from the allocated node as defined by BFAT. Data is read from the node and contained in a buffer (mRNA). After post editing of the pre mRNA, the addition of a 5' cap and poly(A) tail to the mRNA now allows this data package to become part of a dedicated bus. This bus system is local to the insulator cluster node and transfers its message to the ribosome. Each nodal cluster has its own individual bus. It is posited that bus traffic is mediated in part, through the control of the nuclear pore complex responsible for the I/O of the nucleus.

Modeling the cell with a NUMA based architecture allows the multi cellular processing units to have nearly un-limited access to DHD's data base without incurring timing penalties for simultaneous multiple requests. This allows the cell to acquire multiple RNA transcripts without incurring excessive wait states because each transcript is sent on its own independent bus forming a non uniform parallel transmission. RAM access is vastly quicker than node-node communication, but this is an artifact of our current technology based on single processor design/augmentation, which is in turn based on the mechanics/physics of chip design. However, in the cell, no physical prejudice favors a centralized memory storage access structure; in fact, from a robustness and scalability standpoint, a distributed architecture might be advantageous. Part of the justification for this view is that the current generation of HPC machines/platforms are all moving towards the distributive phenotype (BlueGene, PS3 grids, GPU programming). Furthermore, computer scientists are turning to biology as they seek to develop methods for optimizing the use of these distributed computing platforms [43].

### Thermodynamics, dynamic equilibrium, and implications for the design of control systems

It appears that the non-traditional/non-Central Dogma/non-Watson and Crick roles for DNA/RNA seem focused on suppression. In other words, the vast majority of genetic information and cellular resources are spent preventing the activation of established pathways and processes. Cellular information processing, then, seems to be the dependent upon letting up on the "brakes" built into the system. The implications of this viewpoint can be seen in a comparison of the energy states between computers and cells. In computers, the basal state of the computer at rest is "off;" note that no basal energy expenditure is required for the computer to maintain its structure as the actual computer resides in physical space. Once the computer is turned "on" now there is energy input; and information processing can be considered a series of proactive actions; i.e. the energy input goes toward propagating the signal forward. Negative feedback exists, but these exercise their control roles in response to propagating signals through an established circuit. Contrast this with a cell. A cell is never really "off" in terms of energy expenditure, and its physical structure is dependent upon a series of basal dynamic, energy-consuming processes. In short, cells are dissapative thermodynamic systems that exist in a dynamic equilibrium to maintain their high level of organization and structure despite the Second Law of Thermodynamics. This can be interpreted as meaning that the basal condition of the cell is "on," and the laws of thermodynamics would seem to suggest that all the cell's processes must be poised at the "on" position as the cell's organizational structure harbors potential thermodynamic gradients. What keeps all these pathways from executing is inhibition; a series of information processing brakes that are maintained in place. An analogy that comes to mind is the management of a river system with a series of dams and locks; by controlling the various brakes on the tendency of water to flow downhill, the flow is controlled and directed. Therefore there is a critical emphasis on the negative feedback component to biological control. Cellular differentiation (i.e. greater organization) requires a reduction of the possible states of the cell (reducing potential entropy); therefore as the cell matures, greater and greater resources are directed at controlling all the potential processes of inherent to the non-differentiated cell; but that capacity must exist in potential, within the informational content and instruction set, within the stem cell from the beginning! The two far ends of the entropic effects of control can be seen highly relevant areas of biomedical research: at one end the therapeutic use of stem cells requires identifying the correct "boot" sequence to establish the appropriate Bio-OS and subsequent control structure for the mature cell, thereby imposing order onto a potential control structure; at the other end cancer represents an entropic victory, where the control processes maintaining appropriate contextual dynamic equilibrium have degraded.

## Conclusions

In the sections above we have suggested four essential functions of a centralized information-processing complex, and demonstrated how aspects of both a digital computer hard drive and the gene processing machinery of a cell fulfill those criteria. This type of comparison is, admittedly, a very general descriptive framework for a series of very complicated processes, and is subject to the limitations associated with any argument via analogy. However, by providing some degree of mapping between information processes that are, in terms of the digital computer, extensively studied from an engineering standpoint, and processes that are, in terms of cellular control, at the core of a series of pathophysiological processes, we believe that insight can be gained with respect to how and where failures of the system can be identified. As demonstrated in the Discussion Section the multi-functional nature and dynamic adaptability of biological systems imply control properties not currently present in engineered systems. However, by casting these more complex, adaptive characteristics against a defined functional context, it is possible to speculate about the biological components that may serve in those capacities. We believe that the nucleic acids, as described in our comparative framework, fall into this category. The non-coding aspects of RNA and the roles of repetitive DNA have been well-recognized recently, but questions remain about the telos of these properties. However, by recognizing that there must be some internal BFAT and Bio-BIOS for cellular operations, and by mapping the observed actions of RNA to equivalent functions as seen in what would be a "mutable" CHD, these actions can be given a driving context. This allows these processes to be studied as an integrated functional system rather than as a series of isolated molecular activities. This in turn may allow epigenetic mechanisms to be bound together as a higher level of informational syntax. Such information extraction will be essential if we are to be able to "read" our genes, potentially fulfilling the promise offered by learning the "alphabet" by sequencing the human genome.

Our comparative framework between the CHD and the DHD also suggests a cautionary note. It is essential to remember that in terms of the cell as a computer, the message and the medium cannot be separated. This represents an inescapable difference between a computer and a cell. The implication here is that attempts to disrupt the programming of the cell by manipulating its components will invariably lead to unintended consequences. It is also important to note that attempting to reprogram a cell's operations by manipulating its components is akin to attempting to reprogram a computer by manipulating the bits on the hard drive without fully understanding the context of the operating system. The parallel nature of cellular computing reinforces the concept of robustness and redundancy in cellular information processing and function; the flip side of this coin is that in order to affect this machinery significant disruptions and perturbations need to be made, and this degree of intervention is likely to result in a broken machine. Viewed in this fashion, the idea of redirecting cellular behavior by manipulating molecular switches may be fundamentally flawed; that concept is predicated on a simplistic view of cellular computing and control. Rather, may be more fruitful to attempt to manipulate cells by changing their external inputs: in general, the majority of daily functions of a computer are achieved not through reprogramming, but rather the varied inputs the computer receives through its user interface and connections to other machines. This constitutes how the computer operates in a "routine" fashion. It is only when that routine changes such that the existing functionality is no longer sufficient to carry out operations that new programs need to be installed. In the CHD, catastrophic failure may necessitate reformatting, which wipes any existing data, and reinstallation of the operating system. In the CHD, this is possible due to the separation between the medium and the message (though often the hard drive needs to be replaced as well); biological systems do not afford this luxury: we do not have the "next release" of Windows or OS X to install (though stem cell therapy may represent a means of doing this). Rather, we are forced to debug the programs in situ, without crashing the machine. This can only be done by recognizing the control structures involved in cellular information processing and understanding the systems architecture such that therapeutic "patches" can be developed and delivered to correct disordered behavior of the system.

## List of abbreviations used

CHD: Computer Hard Drive; CTs: Chromosome Territories; Qbits: Quad bits; FAT: File allocation system; BFAT: Biological version of FAT; DHD: DNA Hard Drive; ISA: Instruction Set Architecture; BIOS: Basic Input Output System; Bio-BIOS: Biological version of BIOS; OS: Computer Operating System; BioOS: Biological version of operating system; NUMA: Non-Uniform Memory Architecture; RAM: Random Access Memory; ROM: Read Only Memory; SMP: Symmetric Multi-Processing; MAR: Matrix Attachment regions; SAR: Scaffold Attachment Region.

## Competing interests

The authors declare that they have no competing interests.

## Authors' contributions

DD conceived of the initial concept for comparative analysis between the DHD and CHD and and drafted the initial version of the manuscript. GA drafted portions of the manuscript and revised it critically for important intellectual content. All authors read and approved the final manuscript.
